# Supplementary material for: Harnessing machine learning to guide phylogenetic-tree search algorithms
Source: Nat Commun. 2021 Mar 31;12:1983. doi: 10.1038/s41467-021-22073-8 (PMC8012635; doi:10.1038/s41467-021-22073-8)

Supplementary Information

# Harnessing machine learning to guide phylogenetic-tree search algorithms

Azouri et al.

**This PDF file includes:**

Supplementary Figures 1-4
Supplementary Tables 1-4
Supplementary Note 1

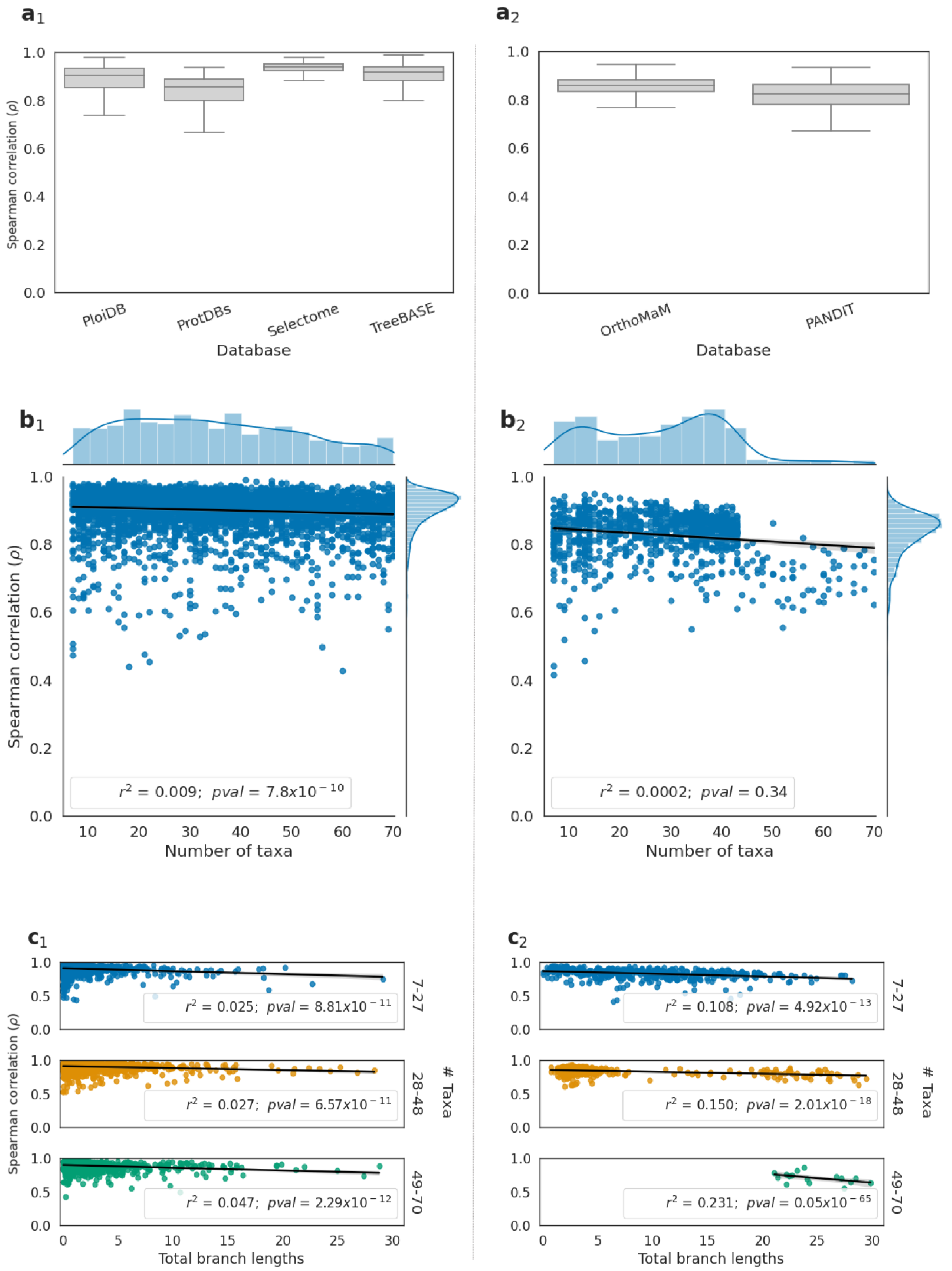

a1
a2
Spearman correlation (ρ)
Database
PloiDB
ProtDBs
Selectome
TreeBASE
OrthoMaM
PANDIT
b1
b2
Number of taxa
r² = 0.009; pval = 7.8x10⁻¹⁰
r² = 0.0002; pval = 0.34
c1
c2
Total branch lengths
# Taxa
7-27
28-48
49-70
r² = 0.025; pval = 8.81x10⁻¹¹
r² = 0.027; pval = 6.57x10⁻¹¹
r² = 0.047; pval = 2.29x10⁻¹²
r² = 0.108; pval = 4.92x10⁻¹³
r² = 0.150; pval = 2.01x10⁻¹⁸
r² = 0.231; pval = 0.05x10⁻⁶⁵

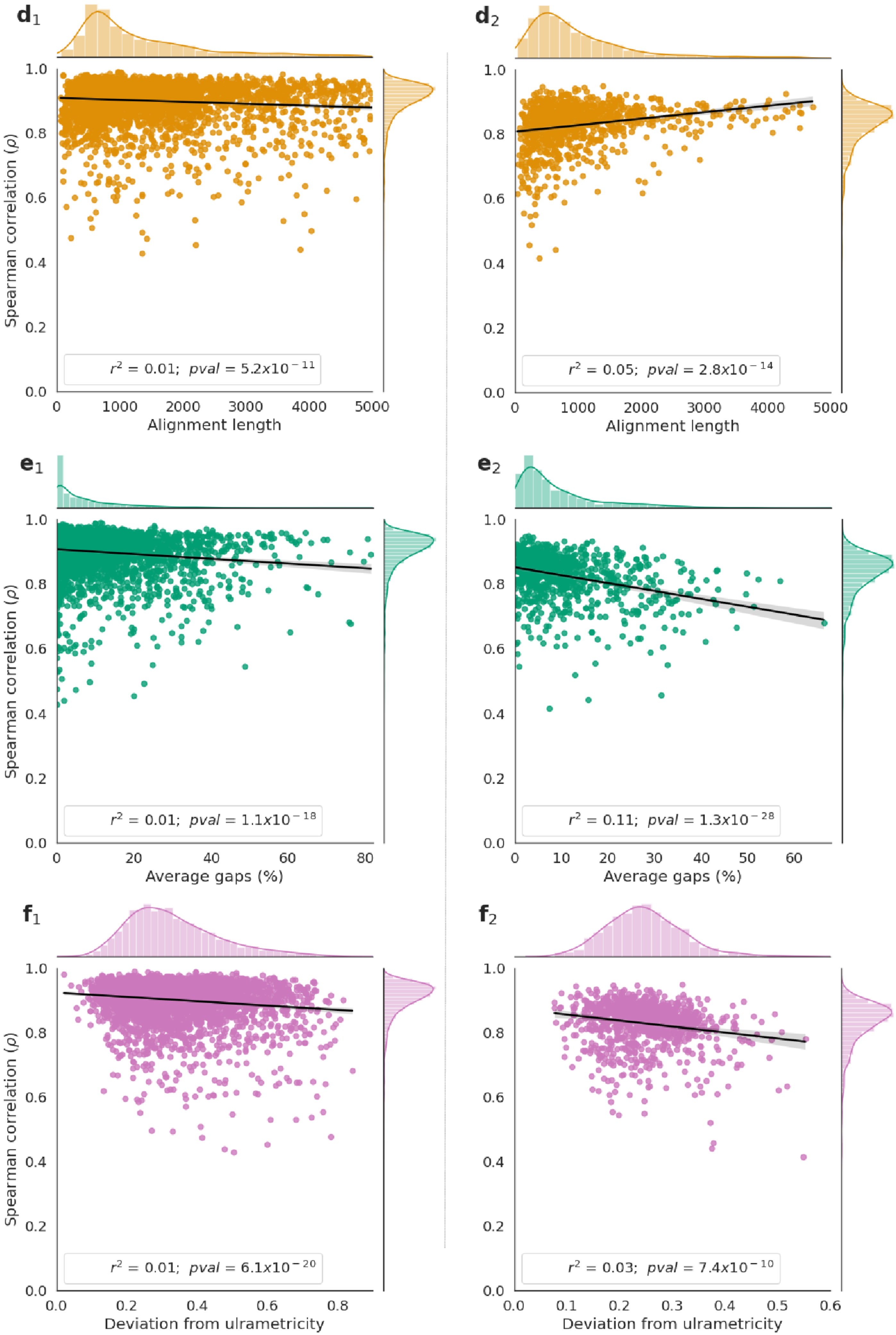

d1
d2
Spearman correlation (ρ)
$r^2$ = 0.01; pval = 5.2x10$^{-11}$
$r^2$ = 0.05; pval = 2.8x10$^{-14}$
Alignment length
e1
e2
$r^2$ = 0.01; pval = 1.1x10$^{-18}$
$r^2$ = 0.11; pval = 1.3x10$^{-28}$
Average gaps (%)
f1
f2
$r^2$ = 0.01; pval = 6.1x10$^{-20}$
$r^2$ = 0.03; pval = 7.4x10$^{-10}$
Deviation from ulrametricity

**Supplementary Figure 1. The dependence between data attributes and Spearman correlation.** The Pearson correlation between model accuracy, as measured by Spearman correlation coefficient ($\rho$), and several data attributes: (a) the six databases used (four for training and two for validation); (b) the number of taxa; (c) the level of divergence as measured by the total branch length; (d) the alignment length; (e) the percentages of gaps in the alignment, averaged across all sequences in the MSA; (f) the deviation from ultrametricity as measured by the MAD score, which quantifies departures from ultrametricity. Left panels represent the training data (4,200 samples), while right panels represent the validation data (1,000 samples). In (a) the box shows the quartiles of the dataset while the whiskers extend to show the 1.5 × IQR past the low and high quartiles, when $n$ is 110, 45, 151, 3,894, 500 and 500 for PloiDB, protDB, Selectome, TreeBase, OrthoMaM and PANDIT, respectively. In (b-f) the '$\mathrm{r}^2$' represents the squared Pearson correlation between the variable in the x axis and the Spearman correlation score of our machine-learning algorithm, and the 'pval' represents the two-sided P value for the null hypothesis that the data are uncorrelated. No adjustments were made for multiple comparisons. The translucent error bands around the regression line is the 95% confidence interval for the regression estimate, calculated using a bootstrap technique.

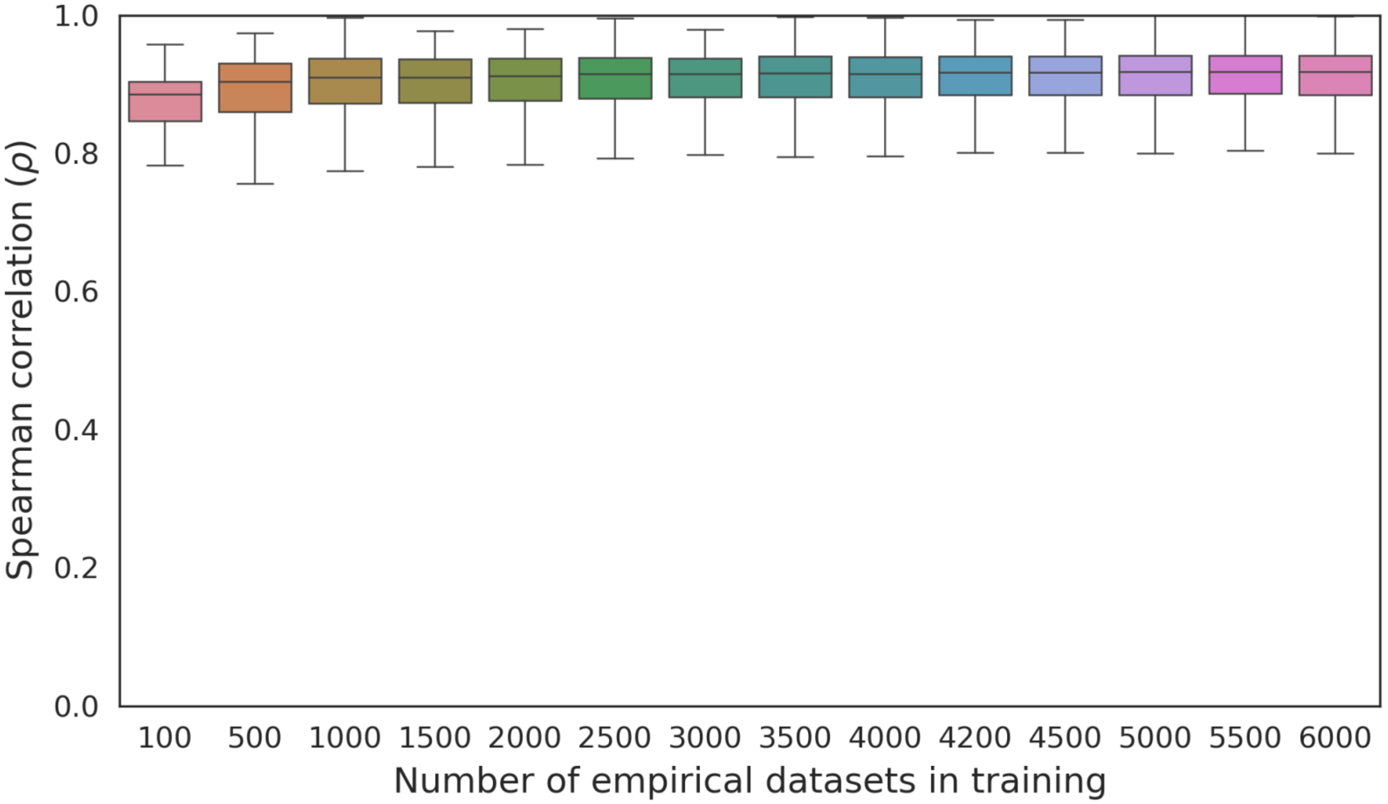


**Supplementary Figure 2. The impact of learning data size on Spearman correlation score.** The mean Spearman correlation coefficient obtained (y axis) when using an increasing number of empirical datasets to generate the training and testing samples for our algorithm (x axis). For each data size, the coefficients are reported as an average of ten 10-fold cross validation iterations, in which a single fold is used for training and the remaining nine for testing. The box shows the quartiles of the dataset while the whiskers extend to show the 1.5 × IQR past the low and high quartiles.

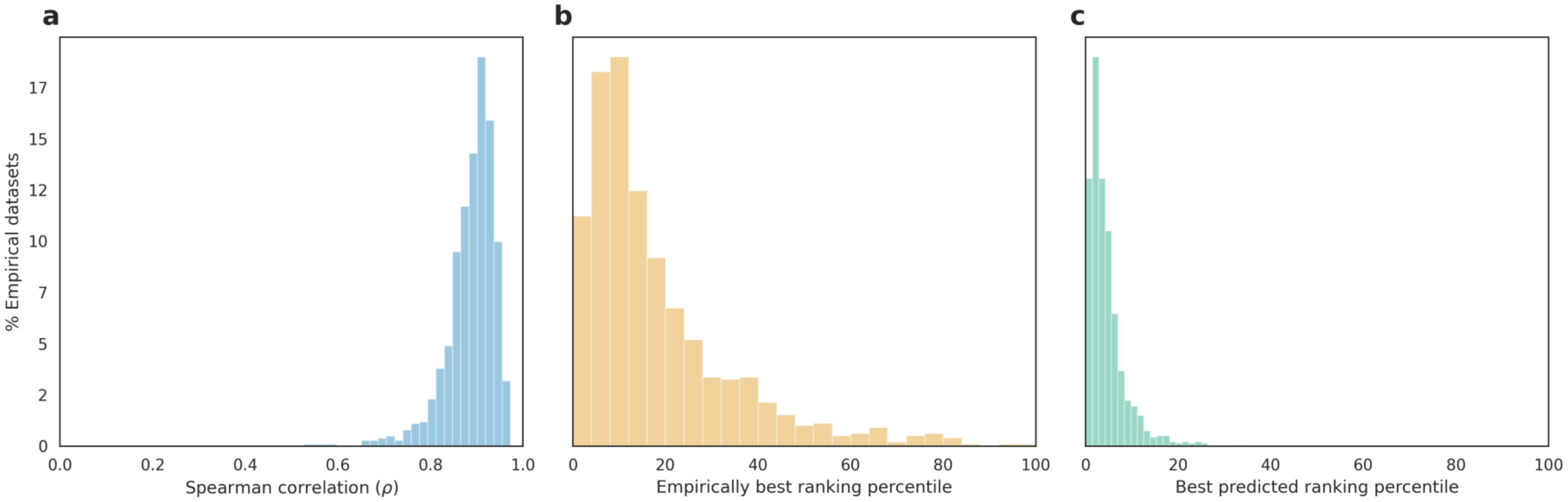


**Supplementary Figure 3. Performance evaluation scores when applying a JC-based-machine-learning model on the validation data.** To train a machine-learning model under the JC mode, we reconstructed NJ starting trees for each of the 4,200 empirical datasets with JC parameter optimization. Next, we calculated the log-likelihood of all neighbors under the JC model. We then trained a machine-learning model to predict the log-likelihood of these neighbors. Finally, we applied the new trained model to the validation data, consists of all neighbors of the 1,000 empirical datasets. The ranking of trees in the validation data was computed based on log-likelihoods under the GTR+I+G substitution model. Histograms for the three performance scores are presented: (a) the Spearman correlation coefficient between the values of the experimentally and the predicted target values; (b) the predicted ranking percentile of the empirically best neighbor; (c) the empirical ranking percentile of the neighbor that was predicted to be the best. On the y axis are the proportions of the empirical datasets (in percentages).

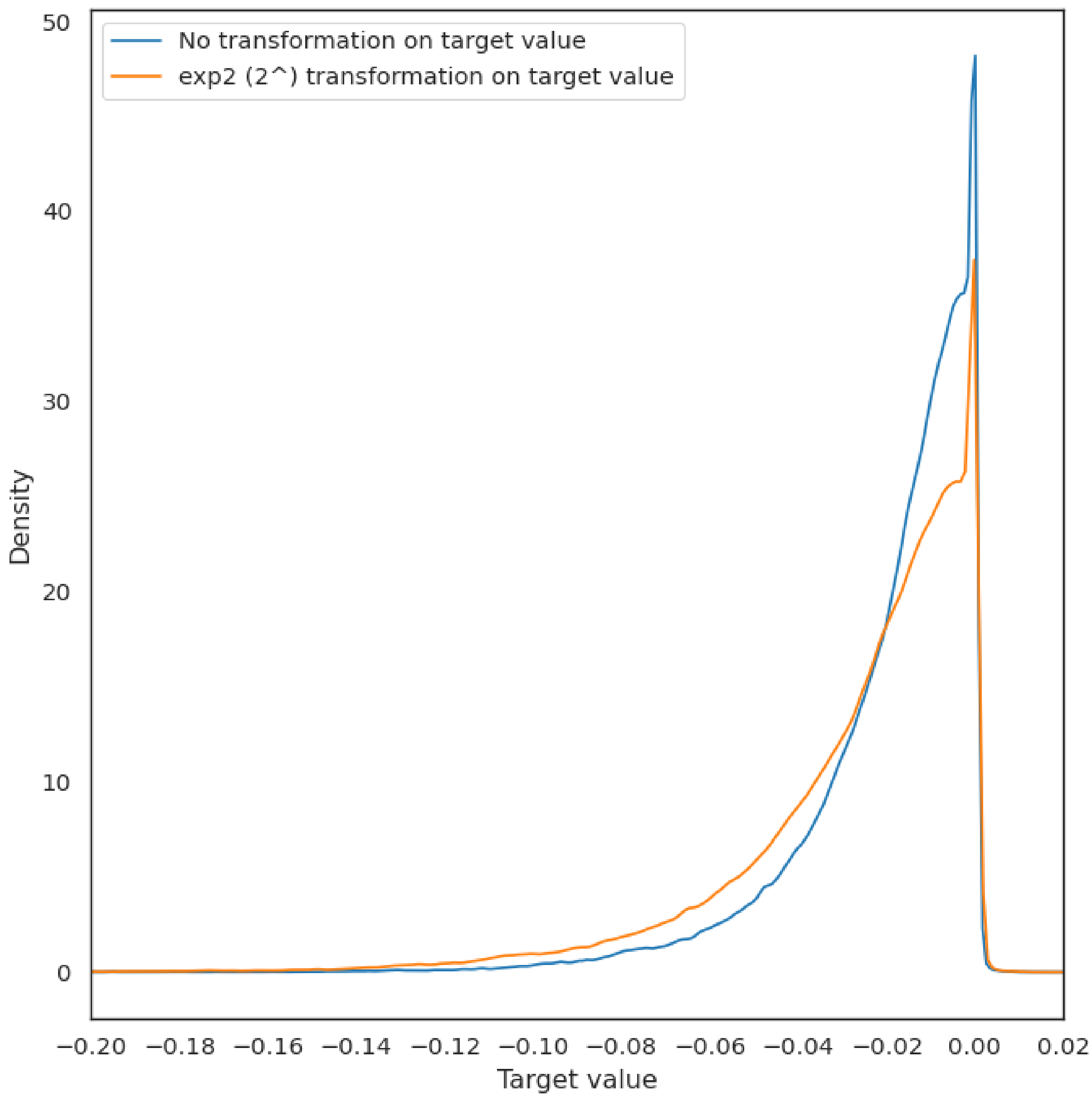


**Supplementary Figure 4. Distribution of the log-likelihood differences of all data points.** Comparison between the target values (log-likelihood difference between a tree and its neighbor, in blue) to a transformed target values, i.e., $f(target) = 2^{target+1}$ (in orange). For interpretability reasons, the transformed values are presented as $f(target) - 2$, i.e., was shifted across the x-axis. The vertical line (in grey) represents the function $x = 0$.

**Supplementary Table 1. Alternative performance evaluation scores when applying a JC-based-machine-learning model on both the testing and validation data.** The accuracy obtained when a machine-learning model was trained under the JC model, as measured by: (i) the percentage of the datasets in which the best move was among the top $t$% predictions; (ii) the percentage of the datasets in which the top-ranked prediction was among the top $t$%. The $t$ variable is defined as the threshold for which we report the scores. The testing data represent the 4,200 datasets that were constructed assuming the JC model, whereas the validation data comprised of 1,000 log-likelihoods computed under the GTR+I+G model.

| Data considered for accuracy evaluation | Metric (i) with $t = 10\%$ | Metric (i) with $t = 25\%$ | Metric (ii) with $t = 10\%$ | Metric (ii) with $t = 25\%$ |
|---|---|---|---|---|
| Testing data | 56% | 88% | 91% | 98% |
| Validation data | 44% | 79% | 90% | 98% |

**Supplementary Table 2. Importance scores of all 19 features used in the machine-learning algorithm.**

| Feature | Importance score (%) |
|---|---|
| The sum of branch lengths in the path between the pruned and the regrafted branches | 34.03 |
| The number of branches in the path between the pruned and the regrafted branches | 9.98 |
| The sum of branch lengths in the starting tree | 9.02 |
| The length of the pruned branch | 7.19 |
| The length of the longest branch of the subtree in Fig. 1b | 7.07 |
| The sum of branch lengths of the subtree in Fig.1b | 4.94 |
| The length of the longest branch in the starting tree | 4 |
| The sum of branch lengths of the subtree in Fig.1c | 3.36 |
| The approximated length of the newly formed branch following pruning | 3.31 |
| The length of the longest branch of the subtree in Fig.1$c_1$ | 2.96 |
| The length of the regrafted branch | 2.44 |
| The length of the longest branch of the subtree in Fig.1c | 2.19 |
| The sum of branch lengths of the subtree in Fig.1$c_2$ | 2.14 |
| The number of leaves in the subtree in Fig.1c | 1.84 |
| The sum of branch lengths of the subtree in Fig.1$c_1$ | 1.77 |
| The length of the longest branch in the subtree in Fig.1$c_2$ | 1.41 |
| The number of leaves in the subtree in Fig.1b | 1 |
| The number of leaves in the subtree in Fig.1$c_2$ | 0.89 |
| The number of leaves in the subtree Fig.1$c_1$ | 0.46 |

**Supplementary Table 3. The accuracy of the machine-learning algorithm when training and testing is performed based on a single feature.**

| Single-feature used in the learning algorithm | Spearman correlation coefficient[a] |
|---|---|
| The sum of branch lengths in the path between the pruned and the regrafted branches | 0.285 |
| The number of branches in the path between the pruned and the regrafted branches | 0.690 |
| The sum of branch lengths in the starting tree | - |
| The length of the pruned branch | 0.141 |
| The length of the longest branch of the subtree in Fig. 1b | 0.140 |
| The sum of branch lengths of the subtree in Fig.1b | 0.011 |
| The length of the longest branch in the starting tree | - |
| The sum of branch lengths of the subtree in Fig.1c | 0.097 |
| The approximated length of the newly formed branch following pruning | 0.067 |
| The length of the longest branch of the subtree in Fig.1$c_1$ | 0.032 |
| The length of the regrafted branch | 0.008 |
| The length of the longest branch of the subtree in Fig.1c | -0.034 |
| The sum of branch lengths of the subtree in Fig.1$c_2$ | -0.062 |
| The number of leaves in the subtree in Fig.1c | 0.036 |
| The sum of branch lengths of the subtree in Fig.1$c_1$ | 0.040 |
| The length of the longest branch in the subtree in Fig.1$c_2$ | 0.121 |
| The number of leaves in the subtree in Fig.1b | -0.072 |
| The number of leaves in the subtree in Fig.1$c_2$ | 0.169 |
| The number of leaves in the subtree Fig.1$c_1$ | 0.039 |

[a]Two features are based on attributes of the starting tree and thus, alone, they cannot discriminate among neighboring trees. For these two features, no Spearman correlation score was computed (represented by '-').

**Supplementary Table 4. The accuracy of alternative supervised-machine-learning regression algorithms.** All algorithms were trained and tested on the same datasets. Spearman correlation coefficients are average over ten 10-fold cross validation procedure.

| Algorithm used | Spearman correlation coefficient |
|---|---|
| Random Forest for Regression (see Methods for hyper-parameters values) | 0.91 |
| Support Vector Machine Regression (using RBF kernel and $epsilon = 5 \times 10^{-6}$) | 0.58 |
| Bayesian Ridge Regression (with default Scikit-learn module hyper-parameters) | 0.61 |
| Lasso Regression (with default Scikit-learn module hyper-parameters) | 0.23 |
| K-Nearest-Neighbors Regression (using $k$ = 5) | 0.21 |

**Supplementary Note 1: Algorithm for efficient feature extraction**

To compute the features of the two trees induced by a separating branch (pruning/regrafting) of all neighbors of a candidate tree, we rooted the starting tree (Fig. 1a) in every branch iteratively and treated the two descendants of the root as the two subtrees induced by the branch (Fig. 1b and c, referred to as subroots hereafter).

To produce the features for all possible locations in an efficient manner, we initialized the desired features over a primary tree and updated the affected nodes of every possible tree re-rooting. Specifically:

1) we generated a primary tree by setting an arbitrary tree tip as an outgroup. This tree was traversed in a post-order manner, i.e., from the leaves towards the root, such that the maximal branch, the sum of branch lengths, and the number of tips in every inclusive cluster were computed according to their descendants.
2) Once the features of the primary tree were initialized, we iteratively rotated the tree by setting a different subtree as the outgroup. Namely, for every branch X-Y:
    a. Y is set as an outgroup, meaning that X and Y are the induced subroots
    b. the features that require update are those in the path between the previous subroot and the parent of Y, due to the rotation of hierarchy

Complexity analysis:

For a tree with n tips, i.e., 2n-3 branches, the feature initialization in step 1 occurs in a recursive manner and requires O(1) operations for each of the inner nodes, and thus O(n) operations overall for this step.

Each update in step 2 requires updating the nodes in the path between the previous subroot and the parent of the becoming subroot, i.e., O(log n) operations. However, step 2 was performed in a preorder traversal (determined ahead according to the primary tree in step 1). In this traversal order, the parent is visited, followed by the left and right children.

i. If the visited node is the left child Y, its parent X was set as the subtree root in the previous iteration, and thus the only update required is for the parent (which becomes its sibling).

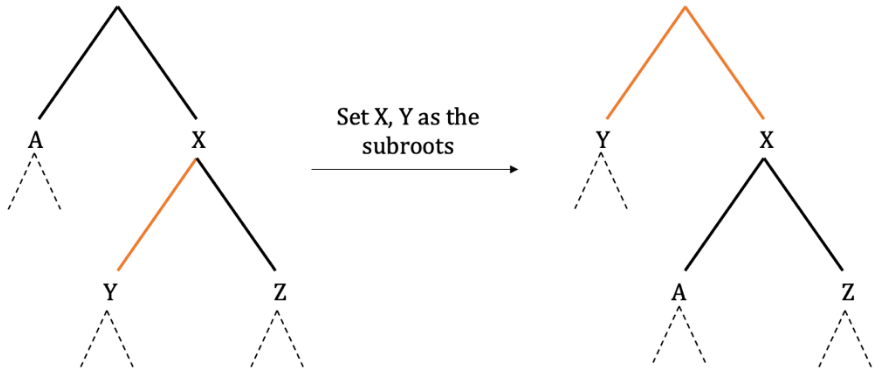


ii. If the visited node is the right child Z, the rightmost descendent of the left sibling was visited and rooted in the previous iteration, and thus the hierarchy in the full path of nodes between them is reversed – every node becomes the parent of its former parent – resulting in O(log n) updated nodes.

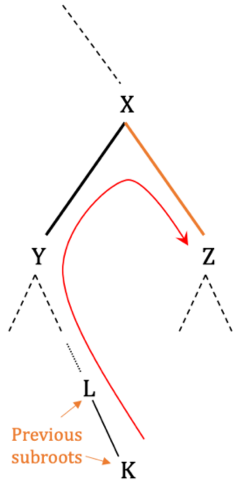


Altogether, for 2n-3 branches, the update requires O(log n) operations, namely, O(nlogn) operations for all the pruning locations of a candidate tree.

**Feature #7 "New branch length" – details**

To estimate the length of the newly branch formed due to pruning ($N_1$,$N_2$), we used a straightforward estimation by simply take the sum of the lengths of the original edges ($Np_1$,$N_1$) and ($Np_1$,$N_2$) before pruning.

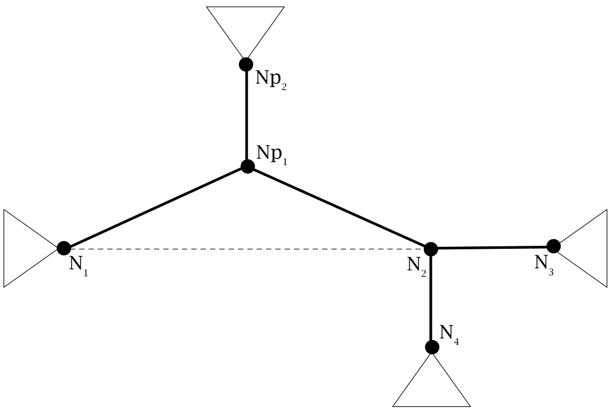

Supplement: Supplementary file 1 — Supplementary Information [file 41467_2021_22073_MOESM1_ESM.pdf]
