# Supplementary material for: Harnessing machine learning to guide phylogenetic-tree search algorithms
Source: Nat Commun. 2021 Mar 31;12:1983. doi: 10.1038/s41467-021-22073-8 (PMC8012635; doi:10.1038/s41467-021-22073-8)
Supplement: Supplementary file 3 — Description of Additional Supplementary Files [file 41467_2021_22073_MOESM3_ESM.pdf]

## Description of Additional Supplementary Files

File Name: Supplementary Data 1
Description: The full list of predicted and true log-likelihood differences obtained from the example dataset for all 2,462 single-step SPR moves. The 'prune_name' and the 'rgft_name' columns represent the node name (either internal or terminal) that defines the prune and regraft actions, respectively. The 'orig_ds_ll' column represents the empirical log-likelihood of the starting tree (same for all rows). The 'll' column represents the empirical loglikelihood of each neighboring tree. The 'dif_ll' column represents the difference between the empirical log-likelihood of each neighboring tree and that of the starting tree. The 'diff_ll_pred' column represents the loglikelihood difference as predicted by the machine-learning algorithm.
