# Supplementary material for: Harnessing machine learning to guide phylogenetic-tree search algorithms
Source: Nat Commun. 2021 Mar 31;12:1983. doi: 10.1038/s41467-021-22073-8 (PMC8012635; doi:10.1038/s41467-021-22073-8)
Supplement: Supplementary file 5 — Reporting Summary [file 41467_2021_22073_MOESM5_ESM.pdf]

nature research

Corresponding author(s): Pupko Tal, Mayrose Itay

Last updated by author(s): Feb 23, 2021

Reporting Summary

Nature Research wishes to improve the reproducibility of the work that we publish. This form provides structure for consistency and transparency in reporting. For further information on Nature Research policies, see our Editorial Policies and the Editorial Policy Checklist.

Statistics

For all statistical analyses, confirm that the following items are present in the figure legend, table legend, main text, or Methods section.

n/a	Confirmed	
☐	☒	The exact sample size (*n*) for each experimental group/condition, given as a discrete number and unit of measurement
☒	☐	A statement on whether measurements were taken from distinct samples or whether the same sample was measured repeatedly
☐	☒	The statistical test(s) used AND whether they are one- or two-sided<br>*Only common tests should be described solely by name; describe more complex techniques in the Methods section.*
☒	☐	A description of all covariates tested
☐	☒	A description of any assumptions or corrections, such as tests of normality and adjustment for multiple comparisons
☐	☒	A full description of the statistical parameters including central tendency (e.g. means) or other basic estimates (e.g. regression coefficient) AND variation (e.g. standard deviation) or associated estimates of uncertainty (e.g. confidence intervals)
☐	☒	For null hypothesis testing, the test statistic (e.g. *F*, *t*, *r*) with confidence intervals, effect sizes, degrees of freedom and *P* value noted<br>*Give P values as exact values whenever suitable.*
☒	☐	For Bayesian analysis, information on the choice of priors and Markov chain Monte Carlo settings
☒	☐	For hierarchical and complex designs, identification of the appropriate level for tests and full reporting of outcomes
☐	☒	Estimates of effect sizes (e.g. Cohen's *d*, Pearson's *r*), indicating how they were calculated

Our web collection on statistics for biologists contains articles on many of the points above.

Software and code

Policy information about availability of computer code

Data collection	The code that was used to collect the data in this study was written in python version 3.6.
Data analysis	The code that supports the findings of this study was written in python version 3.6 and has been deposited in Open Source Framework (OSF) with the identifier DOI 10.17605/OSF.IO/B8AQJ51. Computation of likelihoods and parameter estimates were executed using the following application versions: PhyML 3.0, BIONJ (1997), RAxML-NG 0.9.0.

For manuscripts utilizing custom algorithms or software that are central to the research but not yet described in published literature, software must be made available to editors and reviewers. We strongly encourage code deposition in a community repository (e.g. GitHub). See the Nature Research guidelines for submitting code & software for further information.

Data

Policy information about availability of data

All manuscripts must include a data availability statement. This statement should provide the following information, where applicable:

- Accession codes, unique identifiers, or web links for publicly available datasets
- A list of figures that have associated raw data
- A description of any restrictions on data availability

The datasets contained within the empirical set have been deposited in Open Source Framework (OSF) with the identifier DOI 10.17605/OSF.IO/B8AQJ. These datasets were assembled from the following databases: TreeBase (https://treebase.org/treebase-web/urlAPI.html); Selectome (https://selectome.org/); protDB (https://protdb.org/); PlantDB (DOI: 10.3732/ajb.1500424); PANDIT (https://www.ebi.ac.uk/research/goldman/software/pandit); OrthoMaM (https://orthomam.mbb.cnrs.fr/).

Field-specific reporting

Please select the one below that is the best fit for your research. If you are not sure, read the appropriate sections before making your selection.

☐ Life sciences ☐ Behavioural & social sciences ☒ Ecological, evolutionary & environmental sciences

For a reference copy of the document with all sections, see nature.com/documents/nr-reporting-summary-flat.pdf

Ecological, evolutionary & environmental sciences study design

All studies must disclose on these points even when the disclosure is negative.

Study description	Current algorithms for phylogenetic tree reconstruction utilize various heuristic approaches. Here, we train a machine-learning algorithm over an extensive cohort of empirical data to predict the neighboring trees that increase the likelihood, without actually computing their likelihood. Our analyses suggest that machine-learning can guide tree search methodologies towards the most promising candidate trees.
Research sample	We assembled a training data composed of 4,200 empirical alignments from several databases: 3,894 from TreeBase, 151 from Selectome, 45 from protDB and 110 from PlantDB. TreeBase is a repository of user-submitted phylogenies; Selectome includes codon alignments of species within four groups (Euteleostomi, Primates, Glires, and Drosophila); protDB includes genomic sequences that were aligned according to the tertiary structure alignments of the encoded proteins published in BALIBASE; and PlantDB contains alignments with sequences belonging to a single plant genus and a potential outgroup. We randomly selected datasets with 7 to 70 sequences and more than 50 sites, excluding alignments containing sequences that are entirely composed of gapped or missing characters. To test the predictive power of our model also over unseen validation data that were neither used for training our model nor for cross validation, we gathered a database encompassing 1,000 multiple sequence alignments, collected from two databases that were not used to generate the training set: 500 datasets from PANDIT, which includes alignments of coding sequences, and 500 datasets from OrthoMaM, a database of orthologous mammalian markers. Next, we verified that our validation set is composed of a variety of biological data attributes.
Sampling strategy	Sampling size was determined to produce statistical power and encompass the range of datasets sizes. Datasets were randomly sampled from each database, considering the filtering rules described below in 'Data exclusions'.
Data collection	Data were downloaded from the online databases sources by S. Abadi, sampled and processed using python scripts.
Timing and spatial scale	Not relevant.
Data exclusions	We randomly selected datasets with 7 to 70 sequences and more than 50 sites, excluding alignments containing sequences that are entirely composed of gapped or missing characters.
Reproducibility	Replicates were not relevant to this study as we relied on a large collection of independent datasets to establish our findings.
Randomization	Datasets were randomly sampled from each database, considering the filtering rules described above in 'Data exclusions'.
Blinding	Not relevant - data were obtained from online resources.

Did the study involve field work? ☐ Yes ☒ No

Reporting for specific materials, systems and methods

We require information from authors about some types of materials, experimental systems and methods used in many studies. Here, indicate whether each material, system or method listed is relevant to your study. If you are not sure if a list item applies to your research, read the appropriate section before selecting a response.

Materials & experimental systems

n/a	Involved in the study	
☒	☐	Antibodies
☒	☐	Eukaryotic cell lines
☒	☐	Palaeontology and archaeology
☒	☐	Animals and other organisms
☒	☐	Human research participants
☒	☐	Clinical data
☒	☐	Dual use research of concern

Methods

n/a	Involved in the study	
☒	☐	ChIP-seq
☒	☐	Flow cytometry
☒	☐	MRI-based neuroimaging
